# Supporting implementation of the national standards for cancer survivorship care: development of the cancer survivorship maturity model (CSMM)

**DOI:** 10.1007/s11764-026-02131-0

**Published:** 2026-10-01

**Authors:** Heidi Dowst, Maryem Shirzadi, Martha Mims, Susan Hilsenbeck, Liwei Wang, Hoda Badr

**Affiliations:** 1 Dan L Duncan Comprehensive Cancer Center, Baylor College of Medicine, Houston, TX USA; 2 McWilliams School of Biomedical Informatics,UTHealth, Houston, USA; 3 Department of Medicine, Baylor College of Medicine, Houston, TX USA; 4 Lester and Sue Smith Breast Cancer Center, Baylor College of Medicine, Houston, TX USA; 5 Dell Medical School, The University of Texas at Austin, Austin, TX USA; 6 Department of Medical Oncology, Thomas Jefferson University, Philadelphia, PA USA; 7 Sidney Kimmel Comprehensive Cancer Center, Philadelphia, PA USA; 8 Department of Medical Oncology, Thomas Jefferson University, 834 Chestnut St, Philadelphia, PA 19104 USA

**Keywords:** Cancer survivorship, National Standards for Cancer Survivorship Care, Organizational maturity, Maturity model

## Abstract

**Purpose:**

The National Standards for Cancer Survivorship Care define the domains of care that healthcare organizations should deliver, yet few tools exist to assess organizational capability and implementation maturity for the National Standards. We developed and preliminarily evaluated the cancer survivorship maturity model (CSMM), a framework designed to assess organizational maturity in real-world oncology settings.

**Methods:**

We conducted a mixed-methods, participatory model development study. A preliminary CSMM was refined through five multidisciplinary focus groups at a comprehensive cancer center using consensus-building techniques adapted from nominal group technique. Participants completed prioritization exercises, contributed to model refinement, and assessed the maturity of survivorship care delivery in their practice settings. Qualitative analysis identified model refinement needs and implementation barriers. The final model was distributed to external stakeholders for preliminary evaluation.

**Results:**

Fourteen internal participants and 12 external respondents contributed to model development and evaluation. The final CSMM included seven domains across five maturity levels ranging from ad hoc to optimized. Participants identified differences in prioritization across clinical impact, implementation feasibility, and perceived patient importance. Participants’ assessment of survivorship care maturity was generally low, with most domains clustering between levels 1 and 2. Qualitative findings identified barriers, including unclear workflows, limited resources, insufficient time, and technical constraints.

**Conclusions:**

The CSMM provides a structured approach to assess organizational capability and implementation maturity for the National Standards while identifying domain-specific opportunities for improvement.

**Implications for Cancer Survivors:**

By helping institutions target areas for improvement and resource investment, the CSMM may support more consistent patient-centered survivorship care delivery.

**Supplementary Information:**

The online version contains supplementary material available at https://doi.org/10.1007/s11764-026-02131-0.

## Background

A cancer diagnosis has far-reaching effects that extend well beyond the direct treatment of disease, influencing nearly every aspect of a patient’s physical, emotional, social, and financial well-being. The National Cancer Institute (NCI) defines survivorship as beginning at the time of diagnosis and continuing throughout the balance of life, reflecting the need for coordinated care across the cancer continuum [1]. Survivors frequently experience persistent symptom burden, financial hardship, disruptions in social and occupational functioning, and ongoing needs for health promotion, supportive care, and communication across oncology and primary care settings [1–4]. As the number of cancer survivors continues to grow, healthcare systems are increasingly expected to deliver survivorship care that is comprehensive, coordinated, and responsive to these multidimensional needs.

In response to this need, national organizations including the Institute of Medicine, the NCI, and the American Society of Clinical Oncology have called for survivorship care to become a routine component of high-quality cancer care [1–3]. More recently, the National Standards for Cancer Survivorship Care have provided a more explicit framework for the domains of care that healthcare organizations should be prepared to address, including surveillance, symptom management, supportive services, care coordination, and patient-centered follow-up [1]. These standards represent an important advance for the field because they move survivorship care from a broadly endorsed principle to a more actionable organizational expectation. However, standards alone do not ensure implementation. Healthcare organizations vary substantially in the infrastructure, workflows, staffing models, clinical integration, and data systems needed to operationalize survivorship care consistently across practice settings [5].

A major challenge for the field, therefore, is not only defining what survivorship care should include but also determining whether organizations have the organizational capability to implement it. Although survivorship frameworks and guidelines describe recommended domains of care, fewer tools exist to assess an organization’s implementation maturity across these domains in routine practice.

Several established survivorship quality frameworks and accreditation standards address elements of survivorship care delivery, including Commission on Cancer Standard 4.8 monitoring metrics, the LIVESTRONG Cancer Survivorship Center of Excellence Network indicators, ASCO Quality Oncology Practice Initiative (QOPI) survivorship measures, and National Accreditation Program for Breast Centers (NAPBC) standards [6–9]. These efforts have advanced the field by defining core survivorship services and establishing performance expectations; however, they primarily function as checklists or compliance-oriented metrics and do not provide a structured framework for assessing an organization's stage of implementation across survivorship domains. In contrast, maturity models are widely used in healthcare and informatics to characterize progressive stages of organizational capability, reflecting the extent to which the infrastructure, workflows, staffing, governance, and data systems needed to support implementation are in place [10, 11]. While originally developed to evaluate process capability and organizational performance, maturity models have increasingly been applied in healthcare and biomedical informatics to assess organizational capability for implementation and scale [12, 13]. Their value lies not only in identifying organizational strengths and gaps but also in providing a practical roadmap for staged improvement through domain-specific representations of incremental organizational maturity. In this context, organizational maturity reflects the developmental stage of an organization’s capability to implement the National Standards for Cancer Survivorship Care. Accordingly, the CSMM is intended to assess organizational capability and characterize its maturity to support implementation of the National Standards rather than directly measure the quality of survivorship care. Such a framework can be applied during implementation planning to identify domains requiring additional coordination, training, or resource allocation. To address these practical implementation needs, this study aimed to develop and preliminarily evaluate the cancer survivorship maturity model (CSMM), a clinician-informed framework grounded in the National Standards for Cancer Survivorship Care and designed to assess organizational capability and maturity for implementation of the National Standards in real-world oncology settings. The CSMM characterizes organizational capability across key survivorship domains through progressive levels of implementation maturity that can guide quality improvement and infrastructure development. At the institutional level, such a model may help organizations identify organizational capability gaps and prioritize survivorship care investments. At a broader level, it may help illuminate common implementation barriers and variation in organizational maturity across settings, with implications for dissemination, technical assistance, and future survivorship research. To our knowledge, such maturity-based frameworks have been minimally applied in cancer survivorship, representing a key gap that the CSMM was designed to address.

## Methods

### Study design

We conducted a mixed-methods, participatory model development study to develop and preliminarily evaluate the CSMM, an organizational maturity framework designed to assess organizational maturity for implementation of the National Standards for Cancer Survivorship Care in clinical oncology settings. Early-stage model development was informed by structured focus groups using consensus-building techniques adapted from the nominal group technique (NGT), followed by external stakeholder evaluation for transferability [14–16]. This design was selected to ensure that the model was both conceptually grounded in the National Standards for Cancer Survivorship Care and informed by real-world clinical workflows and implementation contexts. NGT-informed approaches are commonly used in health services research to elicit expert perspectives, reduce dominance by individual participants, and support structured prioritization and refinement of emerging frameworks [17, 18].

### Setting and participants

#### Internal focus groups

Five focus groups were conducted at the Dan L Duncan Comprehensive Cancer Center. Participants were recruited using purposive sampling to capture a range of perspectives across disciplines involved in survivorship care delivery. Eligible participants included multidisciplinary clinicians and supportive care professionals with direct experience in survivorship care pathways, including oncology, primary care, nursing, social work, rehabilitation, and psychosocial services. This approach was used to ensure that the model reflected the interdisciplinary nature of survivorship care implementation. We aimed to enroll 4 to 6 participants per group, consistent with recommended group sizes for focus group and adapted NGT studies [17, 18]. Discussions were moderated by a researcher with expertise in maturity model development (HD) and a second researcher with domain expertise in cancer survivorship care (HB).

#### External stakeholder evaluation

Following internal model refinement, the CSMM was distributed electronically to stakeholders affiliated with the National Cancer Institute Survivorship Supplement Awardees (NCISS) network to obtain preliminary feedback on usability and applicability across institutions. External participants were invited to complete the model as an institutional self-assessment of organizational maturity for implementing the National Standards.

### Development of the preliminary CSMM

A preliminary (“strawman”) version of the CSMM was developed prior to focus group convening using the National Standards for Cancer Survivorship Care as the foundational framework [1]. The goal was to translate these standards into an organizational maturity assessment framework suitable for structured maturity assessment. Because the National Standards define the components of survivorship care that healthcare organizations should provide rather than the organizational capabilities required to implement them, direct one-to-one translation of the 10 standards into a maturity model was not methodologically optimal. Maturity models assess organizational implementation functions rather than individual clinical services and therefore require domains that reflect how survivorship care is operationalized within healthcare systems [10–13, 19, 20]. Accordingly, conceptually related standards that depended on common organizational functions, including shared workflows, staffing models, multidisciplinary coordination, referral pathways, and institutional infrastructure, were consolidated into broader operational domains. This approach preserved alignment with all 10 National Standards while creating a framework that could more accurately assess an organization’s implementation maturity across survivorship domains in routine practice.

**Fig. 1:**
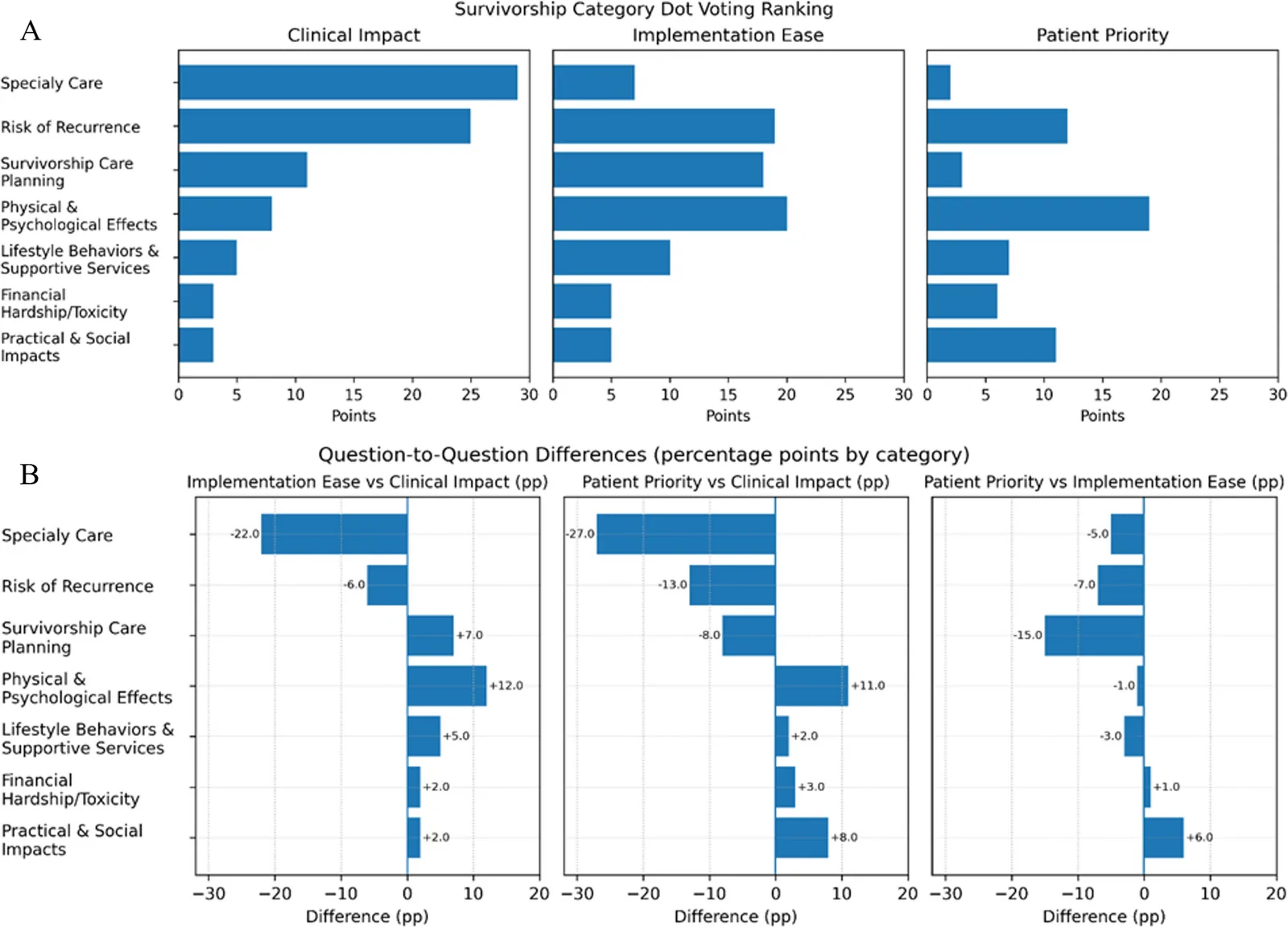
Focus group prioritization of cancer survivorship care domains across clinical impact, implementation ease, and patient priority. **A** Focus group rankings of cancer survivorship categories ordered by perceived clinical impact, with corresponding rankings shown for implementation ease and patient priority. **B** Divergent bar chart displaying absolute percentage-point differences in rankings between question 1 (clinical impact), question 2 (implementation ease), and question 3 (patient priority), highlighting areas of concordance and divergence across prioritization dimensions. CSMM domains were mapped to the National Standards for Cancer Survivorship Care Guideline domains as shown in Footnote^a^. Footnote^a^: *physical and psychological effects* aligns with guideline domains addressing assessment of physical and psychological effects with associated treatment or referrals (National Standards domains 2 and 4); *survivorship care planning* aligns with facilitation of survivorship care and care coordination (domains 1 and 10); *risk of recurrence* reflects assessment of recurrence or new cancers with referral (domain 6); *specialty care* aligns with access to specialty care for late effects (domain 3); *financial hardship/toxicity* maps to assessment of financial hardship and provision of financial support (domain 9); *lifestyle behaviors and supportive services* aligns with assessment of lifestyle behaviors and access to supportive health services (domains 7 and 8); and *practical and social impacts* corresponds to assessment of practical and social needs with provision of resources or referrals (domain 5).

The preliminary CSMM included seven domains that collectively represent all 10 National Standards (Supplemental Table 1): survivorship care planning, risk of recurrence, specialty care, financial hardship/toxicity, lifestyle behaviors and supportive services, practical and social impacts, and physical and psychological effects. Rather than reproducing each National Standard as an individual maturity domain, the CSMM groups standards according to the organizational capabilities required for implementation. For example, the National Standards addressing Lifestyle Behaviors and Supportive Health Services were combined because they are often operationalized through similar screening processes, referral pathways, multidisciplinary supportive care teams, patient navigation, and community partnerships. Likewise, physical and psychological effects were combined because assessment and management of treatment-related symptoms typically occur through coordinated survivorship symptom management processes that require similar organizational infrastructure. Practical and social Impacts were also retained as a single domain because these needs are commonly identified through shared assessment processes and addressed through coordinated navigation, social work, and community resource networks. Organizing the model around implementation capabilities rather than individual standards allowed the CSMM to characterize organizational maturity while preserving conceptual alignment with all 10 National Standards (Fig. 1 Footnote^a^).

Each category was represented across five maturity levels, adapted from established maturity model frameworks [10–13, 19, 20], ranging from level 1 (ad hoc) to level 5 (optimized). Consistent with the maturity model literature, the labels represent progressively higher levels of organizational maturity for implementing the National Standards. Draft descriptors for each maturity level were developed to reflect observable institutional characteristics, including workflows, staffing, referral processes, documentation practices, and use of data to support survivorship care delivery. The preliminary model is provided in Supplemental Table 1.

### Focus group procedures

Each focus group followed a structured process designed to refine model content, assess perceived implementation priorities, and identify organizational barriers affecting implementation of the National Standards. Participants first completed a brief demographic survey capturing clinical role and practice context. The moderator then introduced the study objectives and provided a standardized overview of survivorship care standards and maturity model concepts. Participants completed an initial prioritization exercise using dot voting, a low-burden adaptation of NGT multivoting [17, 18]. They were asked to rank CSMM domains based on three implementation-oriented questions: (1) which domain would have the greatest clinical impact if effectively implemented, (2) which domain would be easiest to implement, and (3) which domain patients would prioritize most highly. This exercise was designed to capture differences between clinical importance, implementation feasibility, and perceived patient-centered priorities.

Following this exercise, the moderator reviewed the structure of the strawman model and the meaning of the five maturity levels to ensure a shared understanding of the framework. Participants then reviewed assigned domains and proposed modifications to the draft maturity level descriptors. The goal of this step was to enhance clarity, face validity, and real-world relevance by ensuring that each level reflected recognizable institutional capabilities and workflows.

Participants reconvened for facilitated group discussion of proposed modifications. Discussions focused on refining domain definitions, resolving ambiguities, and identifying cross-cutting implementation challenges. Focus group discussions were audio recorded, and moderator notes were documented to support model refinement and qualitative analysis. These materials were used to iteratively revise the model. Focus groups were conducted using a pragmatic stopping approach rather than a formal saturation criterion, consistent with prior work highlighting the ambiguity in defining and operationalizing saturation and the importance of aligning sample size decisions with study purpose and analytic approach [21].

Participants then completed a current-state assessment by rating the perceived maturity of their own practice setting for each domain. Aggregate responses were displayed in real time using radar plots to visually summarize domain-level maturity patterns. These visualizations served as a structured reflection prompt, allowing participants to interpret the quantitative ratings, discuss areas of relative organizational strength and weakness, and consider whether observed maturity patterns aligned with the domains they had previously identified as high priority, clinically impactful, or most relevant to patients [22]. These discussions provided qualitative context for the quantitative findings and informed interpretation of the final CSMM.

### Transferability evaluation

Because no established framework for evaluating maturity models has been published, external evaluation is important for assessing the transferability of newly developed models across healthcare settings and reducing the potential for model bias [12]. Following refinement through internal focus groups, the CSMM was implemented in REDCap and distributed to external stakeholders within the NCISS network [23, 24]. Respondents completed the model as an institutional self-assessment of organizational maturity for implementing the National Standards. To enhance interpretability, respondents were shown their individual domain scores alongside aggregated responses from prior participants. External evaluation was intended to provide an initial assessment of the model’s usability, transferability, and ability to capture variation in organizational maturity across institutional contexts.

### Analysis

This study used a mixed-methods analytic approach to integrate structured prioritization data, maturity ratings, and qualitative focus group feedback.

#### Quantitative analysis

Prioritization responses from the focus groups were summarized using weighted rankings. For each prioritization question, first-, second-, and third-place rankings were assigned 3, 2, and 1 points, respectively, and summed across participants to generate domain-level scores. Maturity ratings were summarized descriptively by domain and participant group. Means and standard deviations were calculated for internal and external respondents. Visualizations, including radar plots, were used to summarize domain-level maturity patterns and support interpretation of variation within and between respondent groups. To explore differences in maturity ratings between internal and external respondents, ordinal logistic regression models were conducted for each domain. Given the limited sample size, the quantitative analyses were highly exploratory and intended to descriptively characterize patterns of organizational maturity across domains and participants rather than support formal statistical inference or hypothesis testing.

#### Qualitative analysis

Audio recordings, moderator notes, and documented model modifications from the focus groups were reviewed by the research team using an applied qualitative approach. Analysis focused on two prespecified areas: (1) feedback that informed revision of CSMM domains and maturity level descriptors and (2) recurring implementation barriers affecting delivery of survivorship care. Through iterative review and discussion, the research team identified recurrent patterns and representative quotations that informed refinement of the model and interpretation of quantitative findings.

#### Integration of mixed-methods findings

Qualitative and quantitative data were integrated throughout development and preliminary evaluation of the CSMM. During focus groups, qualitative discussion informed refinement of the CSMM domains and maturity descriptors by identifying areas requiring greater conceptual clarity, operational specificity, and alignment with clinical workflows. Quantitative prioritization exercises and maturity ratings complemented these discussions by characterizing perceived differences in clinical impact, implementation feasibility, patient priority, and organizational maturity across survivorship domains. During the current-state assessment, quantitative maturity ratings were displayed in real time and served as structured reflection prompts, allowing participants to interpret observed maturity patterns and discuss the organizational factors contributing to areas of relative strength and weakness. Together, the qualitative and quantitative findings provided complementary insights that informed refinement and interpretation of the final CSMM and characterized organizational capability and implementation maturity for the National Standards.

## Results

### Participant characteristics

A total of 14 multidisciplinary participants contributed to model development across five internal focus groups. Participants represented a range of disciplines involved in survivorship care delivery, including oncology, primary care, nursing, social work, rehabilitation, and psychosocial services, and practiced across diverse care settings within the cancer center, including academic, safety-net, and Veterans Affairs environments. In addition, 12 external respondents from the NCISS network participated in preliminary external evaluation. Detailed participant characteristics are presented in Table 1.

**Table 1:** Participant characteristics by group.

Characteristic	BCM (*n* = 14)	NCISS (*n* = 12)	Overall (*N* = 26)
**Age (overall)**			
< 30	1 (7.1)	0 (0.0)	1 (3.8)
30–39	4 (28.6)	2 (16.7)	6 (23.1)
40–49	3 (21.4)	5 (41.7)	8 (30.8)
50–59	4 (28.6)	2 (16.7)	6 (23.1)
60–69	1 (7.1)	2 (16.7)	3 (11.5)
70 +	1 (7.1)	1 (8.3)	2 (7.7)
**Gender**			
Female	7 (50.0)	12 (100.0)	19 (73.1)
Male	7 (50.0)	0 (0.0)	7 (26.9)
**Race**			
White	9 (64.3)	11 (91.7)	20 (76.9)
Asian	4 (28.6)	0 (0.0)	4 (15.4)
Black or African American	0 (0.0)	1 (8.3)	1 (3.8)
Other	1 (7.1)	0 (0.0)	1 (3.8)
**Ethnicity**			
Not Hispanic	13 (92.9)	11 (91.7)	24 (92.3)
Hispanic	1 (7.1)	1 (8.3)	2 (7.7)
**Years of clinical experience**			
< 5 years	2 (14.3)	1 (8.3)	3 (11.5)
5–10 years	3 (21.4)	2 (16.7)	5 (19.2)
11–15 years	3 (21.4)	2 (16.7)	5 (19.2)
16–20 years	4 (28.6)	2 (16.7)	6 (23.1)
> 20 years	2 (14.3)	5 (41.7)	7 (26.9)
**Education**			
Doctoral	13 (92.9)	12 (100.0)	25 (96.2)
Masters	1 (7.1)	0 (0.0)	1 (3.8)
**Occupation**			
Oncologist	11 (78.6)	8 (66.7)	19 (73.1)
NP (oncology)	1 (7.1)	0 (0.0)	1 (3.8)
Primary care provider	1 (7.1)	0 (0.0)	1 (3.8)
Psychologist	1 (7.1)	0 (0.0)	1 (3.8)
Palliative care	1 (7.1)	0 (0.0)	1 (3.8)
Researcher	0 (0.0)	3 (25.0)	3 (11.5)

Values are presented as *n* (%)

### Prioritization of survivorship domains

Participants ranked survivorship domains based on perceived clinical impact, ease of implementation, and perceived patient priority (Fig. 1 A). Risk of recurrence, specialty care, and survivorship care planning were perceived as the domains with the greatest potential clinical impact. Specialty care, risk of recurrence, and survivorship care planning were also perceived as the easiest domains to implement. In contrast, physical and psychological effects, risk of recurrence, and financial hardship/toxicity were more often identified as the highest-priority domains from the patient perspective.

Comparisons across ranking dimensions demonstrated only modest alignment, with notable divergence across domains (Fig. 1B). In particular, specialty care was rated highly for clinical impact but less highly for perceived patient priority, whereas domains related to symptom burden and financial hardship were more strongly associated with patient-centered priorities. This pattern was supported by low overall concordance across rankings (Kendall’s *W* = 0.056) and modest pairwise rank correlations. Clinical impact and implementation ease demonstrated moderate positive alignment (Spearman’s rho = 0.527), as did implementation ease and perceived patient priority (rho = 0.505). In contrast, clinical impact and perceived patient priority showed weak negative alignment (rho =−0.252), suggesting that domains perceived as most influential for clinical outcomes were not always those perceived as most important to patients.

### Qualitative refinement of the cancer survivorship maturity model

Focus group discussions informed iterative refinement of the CSMM, resulting in clarification of domain definitions and maturity-level descriptors. Supplemental Table 1 presents the initial strawman model, Supplemental Table 2 summarizes focus group modifications, and Supplemental Table 3 reflects the final revised CSMM used for external evaluation. Participants generally endorsed the overall seven-domain structure but identified areas of conceptual overlap and ambiguity that required refinement. In particular, participants noted that practical and social challenges often intersect with financial hardship, reflecting shared underlying drivers such as employment disruption and treatment-related costs. While participants did not recommend combining these domains, they emphasized the need for clearer operational distinctions to support accurate assessment. As one participant noted, “practical and social issues bleed into financial issues,” underscoring the importance of distinguishing these domains while acknowledging their interrelated nature. Similarly, participants discussed whether psychological effects should be separated from physical symptom management, citing differences in clinical ownership and referral pathways. Ultimately, participants supported maintaining integrated domains with clearer descriptors that reflected how these issues are addressed in practice.

Across focus groups, participants emphasized that maturity level descriptions should reflect observable institutional processes, including role delineation, referral workflows, and availability of resources. Revisions focused on improving clarity, reducing ambiguity, and ensuring that each level corresponded to recognizable organizational capabilities. Later focus groups yielded few additional modifications, suggesting that the model had stabilized and that a pragmatic stopping point had been reached. For example, when asked by the moderator to provide feedback, one participant in the final focus group remarked, “I don’t have many changes to suggest; it looks pretty good.”

### Internal current-state maturity assessment

**Fig. 2:**
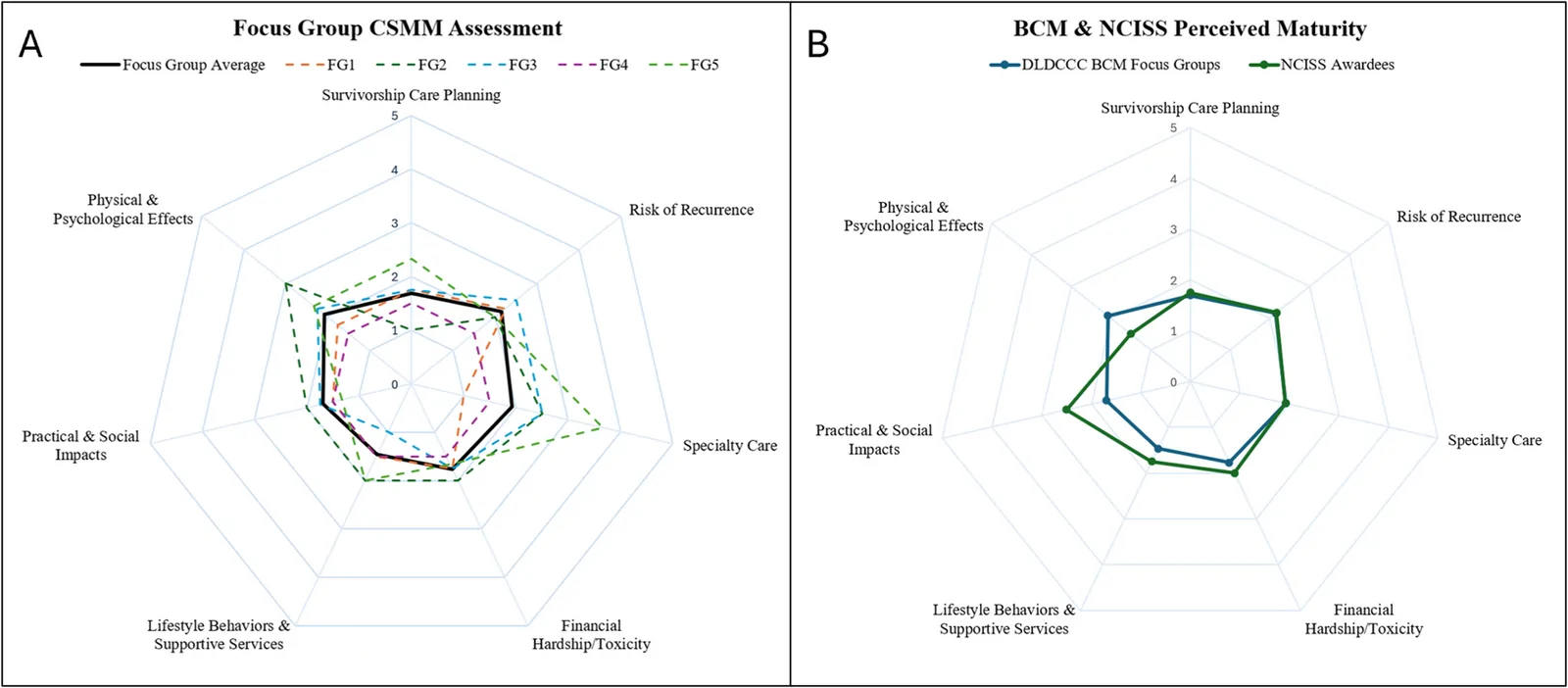
Organizational maturity ratings across the seven CSMM domains. **A** Radar plot displaying current-state maturity ratings for BCM participants. Individual pariticpant ratings are shown as colored dashed lines, and the mean BCM focus group rating is shown as a solid black line. **B** Mean organizational maturity ratings for BCM participants (blue; *n* = 14) and external NCISS respondents (green; *n* = 12). Larger polygons indicate higher organizational maturity across domains.

Participants applied the CSMM to assess the maturity of survivorship care delivery within their primary practice settings (Table 2 and Fig. 2 A). Across domains, maturity ratings were generally low to moderate, with most domains clustering between levels 1 (ad hoc) and 2 (planned), indicating limited standardization and integration of survivorship care processes. Among internal respondents, risk of recurrence and specialty care demonstrated relatively higher average maturity compared with other domains, whereas lifestyle behaviors and supportive services and practical and social impacts were among the lowest-rated areas. These findings suggest that surveillance and referral processes may be more established, while broader supportive care services remain less systematically implemented.

**Table 2:** Internal Participant CSMM Maturity Ratings By Domain. Columns C1 through C14 represent individual BCM participants. The final column presents the mean rating among all BCM respondents.

Focus Group CSMM Assessment	C1	C2	C3	C4	C5	C6	C7	C8	C9	C10	C11	C12	C13	C14	BCM Avg
Specialty Care	1	1	1	1	2	3	2	4	2	2	1	2	4	3	2.07
Risk of Recurrence	2	2	3	2	2	2	2	4	2	2	1	2	2	2	2.14
Survivorship Care Planning	1	1	1	4	1	1	1	2	2	2	1	2	2	3	1.71
Physical & Psychological Effects	1	1	1	4	3	3	2	2	3	2	1	2	2	2	2.07
Lifestyle Behaviors & Supportive Services	1	1	1	3	2	2	1	1	1	1	1	2	2	2	1.50
Financial Hardship/Toxicity	1	2	2	2	1	3	1	2	1	3	1	2	2	2	1.79
Practical & Social Impacts	1	1	2	2	2	2	1	2	2	2	1	2	1	2	1.64

Variation in maturity ratings across focus group participants was observed, particularly in domains such as Specialty Care, which ranged from a mean of 1.00 in one focus group to 3.67 in another. This variability was also reflected in the relatively larger standard deviation for specialty care compared with other domains (Table 3), suggesting meaningful differences in survivorship care infrastructure across clinical settings. Radar plot visualization further demonstrated variability across domains and settings, highlighting uneven development of survivorship care capabilities within the institution (Fig. 2 A).

### External evaluation of CSMM transferability

External respondents from the NCISS network completed the CSMM as an institutional self-assessment of organizational maturity for implementing the National Standards (Table 3 and Fig. 2B). Overall, patterns of domain-level maturity were similar between internal and external groups, with mean scores in both groups remaining in the lower range of the maturity scale. The overall mean maturity rating was 1.85 among internal respondents and 1.93 among external respondents, representing a difference of only 0.08 points on the 5-point scale.

**Table 3:** Mean CSMM maturity ratings by domain and repondent group. Means and standard deviations are presented separately for BCM participants, NCISS respondents, and the combined sample.

Category	Institution	Mean	SD	N
Specialty care	BCM	2.071	1.072	14
	NCISS	1.917	0.793	12
	Overall	2	0.938	26
Risk of recurrence	BCM	2.143	0.663	14
	NCISS	2.083	0.793	12
	Overall	2.115	0.711	26
Survivorship care planning	BCM	1.714	0.914	14
	NCISS	1.75	0.754	12
	Overall	1.731	0.827	26
Physical and psychological effects	BCM	2.071	0.917	14
	NCISS	1.5	0.522	12
	Overall	1.808	0.801	26
Lifestyle behaviors and supportive services	BCM	1.5	0.65	14
	NCISS	1.75	0.866	12
	Overall	1.615	0.752	26
Financial hardship/toxicity	BCM	1.786	0.699	14
	NCISS	2	0.603	12
	Overall	1.885	0.653	26
Practical and social impacts	BCM	1.643	0.497	14
	NCISS	2.5	0.674	12
	Overall	2.038	0.72	26

**Fig. 3:**
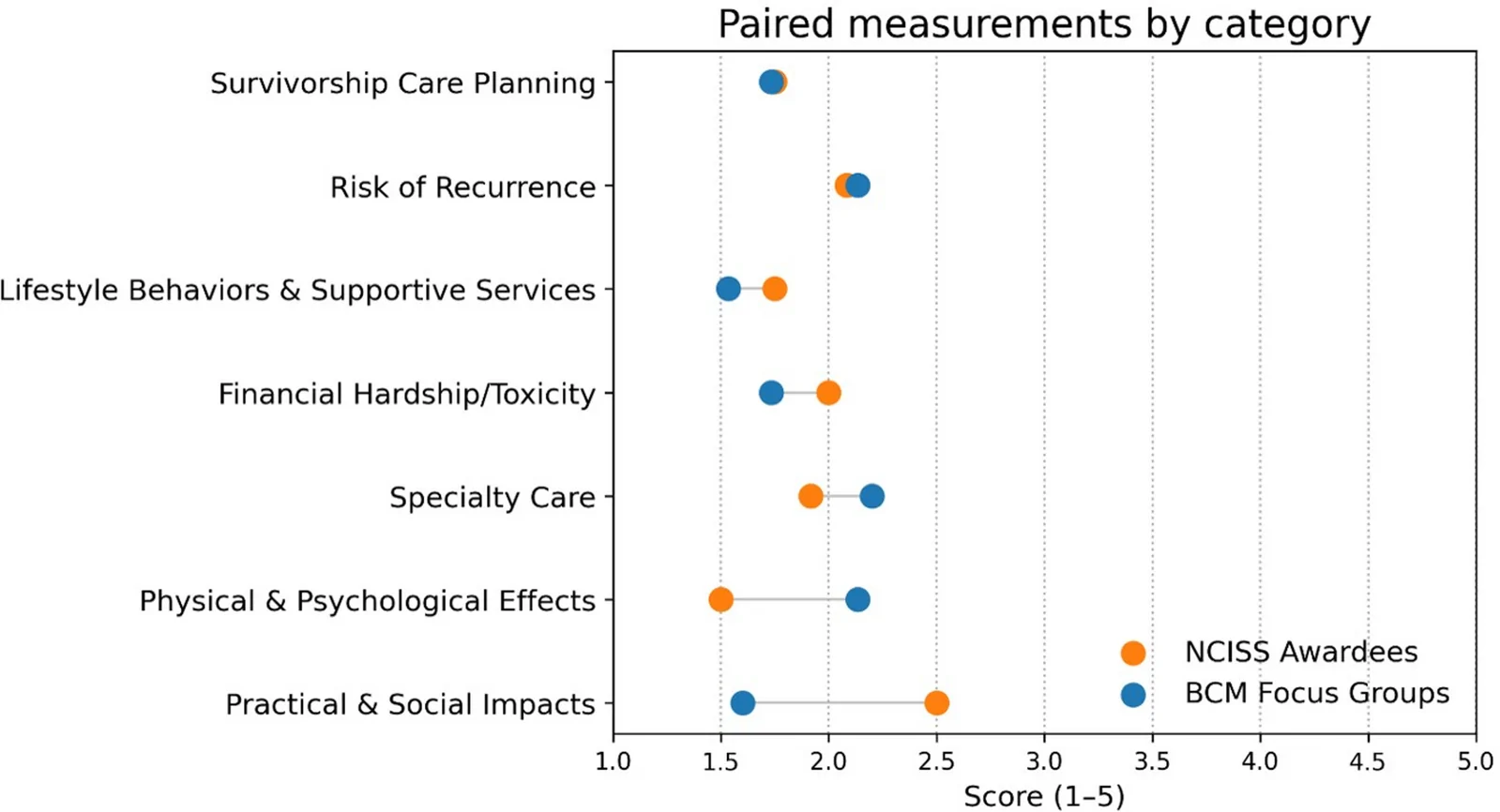
Visualization of mean score differences between BCM and NCISS respondents across cancer survivorship care domains. Survivorship care planning and risk of recurrence show the least between-site variation, whereas practical and social impacts and physical and psychological effects demonstrate the greatest between-site differences. Across all categories, between-site variation is small relative to the full evaluation scale.

Across domains, practical and social impacts was rated relatively higher among external respondents, whereas physical and psychological effects and lifestyle behaviors and supportive services remained among the lowest-rated domains. Comparative visualization demonstrated close alignment between internal and external respondents in most domains, with only modest differences observed (Fig. 3).

Exploratory ordinal logistic regression analyses indicated limited evidence of systematic differences between internal and external groups across domains (Table 4). Although practical and social impacts showed a statistically significant difference between internal and external respondents, interpretation is limited by small sample sizes and wide confidence intervals. Overall, these findings suggest that the CSMM is interpretable and applicable across institutional contexts and may capture common patterns of organizational maturity across survivorship care settings.

**Table 4:** Ordinal logistic regressions (BCM vs. NCISS). Ordinal logistic regression results comparing CSMM ratings between  BCM and NCISS group model assessments by cancer survivorship category. Categories are ordered by *p*-value. Practical and social Impacts was the only domain showing a statistically significant between-group difference.

Category	Total	BCM N	NCISS N	OR NCISS vs. BCM	95% CI	*p*-value
Practical and social impacts	26	14	12	24.58	2.46–246.05	0.0064
Physical and psychological effects	26	14	12	0.26	0.06–1.24	0.0917
Financial hardship/toxicity	26	14	12	2	0.43–9.34	0.3781
Lifestyle behaviors and supportive services	26	14	12	1.71	0.38–7.64	0.4829
Survivorship care planning	26	14	12	1.29	0.30–5.53	0.7323
Risk of recurrence	26	14	12	0.7424	0.1313–4.1983	0.7362
Specialty care	26	14	12	0.8701	0.2125–3.5629	0.8465

### Cross-cutting implementation themes

Qualitative analysis of focus group discussions identified several recurring barriers to survivorship care implementation (Table 5). These included limited availability of institutional resources, insufficient clinician time, lack of technical infrastructure, unclear processes and role delineation, and variation in survivorship care needs across cancer types and clinical settings. Among these, lack of clearly defined processes and responsibilities was the most frequently cited barrier (five mentions), followed by clinician time constraints (4), variation in care across cancer types and settings (3), limited resources (3), and technical barriers (2). Cost or funding constraints were also noted, although less frequently (1). Participants described challenges related to fragmented workflows, lack of ownership of survivorship care tasks, and limited integration of electronic health record systems across care settings. As one participant noted, “there is a lack of workflow clarity about the process and roles,” highlighting the importance of clearly defined responsibilities in survivorship care delivery. Participants also emphasized that survivorship care is resource-intensive and often not supported by dedicated funding or protected time, limiting the ability of clinicians to implement recommended care components.

**Table 5:** Implementation barriers identified through focus group discussions.

	Reflections on barriers to implementation of cancer survivorship care	Themes
FG1	• Survivorship care is specific to each cancer diagnosis • Survivorship care is more resource intensive than the group had initially thought • “Lack of workflow clarity, about the process and roles” • “Discontinuity of EHR systems across clinical practices”	Variation Time Process Technical
FG 2	• “Survivorship care needs vary across cancer type and clinical settings. It is difficult for an institution to provide a one-size-fits-all solution to support it.” • “Survivorship care is unfunded which translates into having no time reserved for these activities with the patient. Manpower is needed to successfully implement cancer survivorship guidelines.” • “Clinician is bombarded with paperwork; screening information should be direct to patient, like a QR code in the lobby.”	Variation Cost and time Process
FG 3	• “Care planning is multidisciplinary, personalized and complex. Time is a limiting factor.” • “A proactive survivorship checklist would be helpful. I like the idea of screening tools but do not box the physician in so that they have no flexibility or send too many EHR alerts causing alert fatigue." • “Physical effects of cancer, follow-ups and referrals all need to be addressed by the physician, but the remaining survivorship axes could be handled by a case manager.” • “More automated screenings are needed for survivorship care, but the institution all needs resources to act upon the data.”	Variation and time Technical Process Resource
FG 4	• “The financial category is one of the ones that the patients are most concerned with and it's also the only one (category) that's at a level one for both of us (oncologist raters). And the reason why it doesn’t get done at a higher level is because it requires having specialty people who understand the resources and can counsel them.” • I think primary care should be handling some of the (cancer survivorship care) categories. I don’t know if I'm necessarily the person who's supposed to be doing it all	Resource Process
FG 5	• “That's always my concern with a lot of these guidelines, that they say, “Oh, you need to do this, that, and the other,” and the institution doesn’t have the people or the resources to actually follow through.” • “Survivorship care has to be personalized to the patient. And it’s complex, but it’s very important. It should be multidisciplinary. It needs to be a proactive plan rather than planning as you go. But we don’t want to box the physician in, and we don’t want to send them a bunch of alerts that they must comply with if it’s not appropriate. And then it would be nice if there were some kind of preference list, but that could be done by a case manager or an intake nurse. There’s a time limitation with the oncologist, so symptoms and side effects, referrals, and follow-ups should be handled by the oncologist, but the rest could be handled by the case manager.”	Time & Resource Process

## Discussion

In this mixed-methods study, we developed and preliminarily evaluated a cancer survivorship maturity model (CSMM) to assess organizational maturity for the National Standards for Cancer Survivorship Care. Across internal and external respondents, organizational maturity was generally low, with most domains clustering between the ad hoc and planned levels of implementation. Participants also identified meaningful differences in how survivorship domains were prioritized depending on whether they were viewed through the lens of clinical impact, implementation feasibility, or perceived patient importance. Qualitative findings further highlighted recurring organizational barriers, including unclear workflows, limited resources, insufficient time, and fragmented systems. Taken together, these findings suggest that while survivorship care is widely recognized as a priority, many healthcare organizations lack the organizational capability and infrastructure needed to implement the National Standards consistently.

One of the most informative findings in this study was the divergence observed across domain prioritization. Domains perceived by respondents as having the greatest potential clinical impact were not always those perceived as easiest to implement or most important to patients. This distinction is important because survivorship care implementation often requires institutions to make practical decisions about where to invest limited resources. Our findings suggest that survivorship program development may benefit from considering multiple implementation lenses simultaneously, including expected impact on clinical outcomes, operational feasibility, and responsiveness to survivor priorities. In this sense, the prioritization exercise may have value beyond model development by offering institutions a structured approach to survivorship care planning and resource allocation.

Across both internal and external respondents, maturity ratings were tightly clustered around the lower end of the five-level scale, with overall mean ratings approximating level 2. This pattern suggests that represented organizations may have foundational survivorship care elements in place, such as individual clinician practices, isolated referral pathways, or basic documentation supports, but may not yet have standardized, integrated, and reportable survivorship workflows. This interpretation is particularly important in the context of the National Standards for Cancer Survivorship Care, which articulates a comprehensive vision for survivorship delivery but may exceed the operational capacity of many institutions in their current state. Risk of Recurrence was the most mature domain, indicating that institutions remain more capable of delivering clinically embedded surveillance activities than implementing comprehensive psychosocial, lifestyle behavioral, and practical survivorship services.

These findings highlight a key implementation gap in cancer survivorship care: the National Standards define what survivorship care should include, but less guidance exists regarding how healthcare organizations can assess their organizational capability and maturity for implementation. The CSMM was designed to address this gap by translating the National Standards into an organizational maturity framework that institutions can use to identify strengths, capability gaps, and staged opportunities for improvement. Rather than functioning as a checklist of services, the model is intended to help organizations assess whether they have the workflows, staffing, referral pathways, documentation practices, and infrastructure needed to implement the National Standards consistently. In this way, the CSMM may be useful not only for internal quality improvement, but also for informing institutional planning, implementation strategy, and future survivorship-focused funding and technical assistance efforts. By distinguishing between domains at earlier versus more advanced stages of organizational maturity, the CSMM may also help institutions prioritize limited resources, implementation efforts, and infrastructure investments needed to strengthen survivorship care delivery over time.

The qualitative findings provide additional insight into how institutions might use the model in practice. Participants frequently described implementation barriers that were organizational rather than conceptual, including unclear ownership of survivorship tasks, lack of technical integration, and insufficient time to address survivorship needs during clinical encounters. Importantly, participants also identified practical opportunities for improvement, such as embedding patient-reported needs screening into clinic workflows and creating more reliable referral triggers for supportive services. These examples suggest that organizational maturity may be advanced not only through additional resources, but also through better workflow design, role clarity, and systems integration. As such, the CSMM may be especially useful as a planning tool for identifying where survivorship care processes break down and where implementation supports are most needed.

### Practical application of the CSMM

Maturity models are traditionally used to support strategic planning, implementation planning, resource allocation, and identification of opportunities for organizational growth. In this context, the CSMM is intended as a structured organizational self-assessment for institutions seeking to implement or strengthen alignment with the National Standards for Cancer Survivorship Care. Primary users may include survivorship program leaders, clinicians, administrators, and other stakeholders involved in survivorship care delivery.

Organizations determine their maturity by comparing their current practices with the descriptors in each domain and selecting the level that most closely reflects their current state. The CSMM is not intended to generate a fixed score or consensus rating. Rather, when completed by multiple stakeholders, differences in ratings provide valuable insight into variation in organizational perspectives across clinical, administrative, and operational roles. These differences should be interpreted as opportunities to facilitate discussion, identify areas requiring organizational alignment, and prioritize implementation efforts rather than as precise differences in organizational maturity.

The next higher maturity level provides practical guidance by identifying the additional characteristics, processes, or resources needed to advance organizational maturity within a given domain. At the end of the online assessment, users receive a radar plot showing their organization’s maturity profile across domains, overlaid with the aggregate community average for comparison. The CSMM may be administered at baseline and repeated over time to monitor progress and refine implementation priorities. Additional educational materials and the online assessment are available for organizations implementing the National Standards at https://redcap.link/CSMM.

### Strengths and limitations

This study has several strengths. First, the CSMM was developed using a participatory, mixed-methods process that incorporated multidisciplinary perspectives from clinicians and supportive care professionals directly involved in survivorship care delivery. Second, the model was refined iteratively through structured focus groups and then applied externally across institutions, providing preliminary evidence of transferability beyond the originating cancer center. Third, integration of qualitative and quantitative findings allowed us to evaluate not only domain-level maturity ratings but also the organizational factors that may shape survivorship care implementation in practice.

This study also has important limitations. First, the CSMM was developed from the National Standards for Cancer Survivorship Care but necessarily consolidated some standards into broader operational domains in order to create a usable maturity model. Although this approach was intended to reflect how survivorship care is operationalized within healthcare systems and facilitate assessment of organizational capability and implementation maturity, it may have reduced granularity for some individual survivorship care elements. Second, the internal focus groups were conducted within a single cancer center, and the external evaluation sample was modest. Notably, the external evaluators were drawn from NCI survivorship supplement-funded institutions, centers that are among the most engaged in survivorship care nationally and would be expected to demonstrate relatively high organizational maturity to implement the National Standards. That maturity ratings nonetheless clustered at early stages in this group is a striking finding, suggesting persistent gaps in organizational capability even in survivorship care-focused settings. Consequently, these scores should not be interpreted as representative of broader oncology practice, as the external evaluation was intended to assess CSMM transferability rather than estimate organizational maturity across cancer centers nationally.

Third, because the purpose of this phase of model development was to characterize organizational capability and implementation maturity for the National Standards, participants were intentionally selected based on their expertise in survivorship care delivery and implementation rather than lived experience as cancer survivors. Consequently, prioritization of patient needs reflects clinician perceptions rather than direct survivor perspectives and should be interpreted accordingly. Future refinement of the CSMM would benefit from incorporating survivors and caregivers to evaluate whether the domains, maturity descriptors, and implementation priorities adequately reflect survivor experiences and priorities, thereby strengthening the model’s face validity and practical relevance. Future research should also evaluate the CSMM across a broader range of oncology practice settings and examine its utility for benchmarking organizational maturity over time, guiding implementation strategies, and informing survivorship care quality improvement efforts. Inclusion of patient and caregiver perspectives will also be important for ensuring that future iterations of the model reflect survivor priorities more directly. As the National Standards continue to evolve, tools that help organizations assess organizational capability and implementation maturity will be increasingly important for translating national guidance into routine clinical practice.

## Conclusion

As the National Standards for Cancer Survivorship Care become more clearly defined, the challenge for cancer centers and healthcare organizations is shifting from defining what survivorship care should include to developing the organizational capability needed to implement those standards consistently and comprehensively. The CSMM offers a practical framework for assessing organizational maturity across key survivorship domains while identifying where organizational infrastructure remains underdeveloped. By helping institutions distinguish areas of relative strength from areas requiring greater investment, the CSMM may support more strategic implementation planning, targeted resource allocation, and broader advancement of survivorship care delivery.

## Supplementary Information

Below is the link to the electronic supplementary material.

ESM 1 (DOCX 60.5 KB)

## Data Availability

The data that support the findings of this study are available from the corresponding author upon reasonable request.
