# Supplementary material for: Supporting implementation of the national standards for cancer survivorship care: development of the cancer survivorship maturity model (CSMM)
Source: J Cancer Surviv. 2026 Oct 1;20(5):1868–81. doi: 10.1007/s11764-026-02131-0 (PMC13723664; doi:10.1007/s11764-026-02131-0)
Supplement: Supplementary file 1 [file 11764_2026_2131_MOESM1_ESM.docx]

**Supplemental Materials**

Supplement Table 1. Pre-Focus Group IMM Model

Cancer Survivorship Maturity Model: Strawman IMM model built by research team prior to clinical focus groups convening based on National Guidelines for Cancer Survivorship Care key domains.

| Level 5 Optimized | Survivorship Care Planning | Risk of Recurrence of New Cancers | Care Referrals | Financial Hardship/Toxicity | Lifestyle Behaviors & Supportive Health Services | Practical and Social Effects | Physical Effects |
| --- | --- | --- | --- | --- | --- | --- | --- |
| Definition:  Care is personalized, proactive, and predictive, supported by AI-driven decision support, real-time data monitoring, and continuous learning systems. Institutions use advanced analytics and closed-loop systems to tailor care to individual patients, deploy resources efficiently, and adjust interventions dynamically based on feedback and emerging evidence. | Advanced analytics enable real-time monitoring of survivorship care plan completion and utilization. AI-driven alerts support personalized and proactive care planning, tailoring recommendations to individual patient needs and clinical trajectories. Workflows are continuously refined by leadership based on outcome data, patient feedback, and evolving best practices, ensuring that SCPs are fully integrated across oncology and primary care. | AI-driven analytics identify high-risk patients for recurrence, enabling personalized and dynamic surveillance plans. Screening intervals and outreach strategies are adjusted in real time based on individual risk profiles and clinical data. Integration with Health Information Exchanges (HIEs) ensures that procedures performed outside the system are captured, allowing for comprehensive care tracking and closing the loop on surveillance adherence. | Predictive analytics identify patients likely to benefit from specialty referrals based on risk profiles, treatment history, and symptom trajectories. Clinicians receive real-time decision support with prioritized referral recommendations, facilitating timely and appropriate care. Referral completion is monitored through closed-loop systems, allowing for continuous refinement of referral pathways and improved coordination across care teams. | Real-time dashboards monitor financial distress during routine cancer clinic visits, using risk models to flag patients at high risk for cost-related nonadherence. Proactive financial counseling interventions are triggered before hardships affect treatment continuity. Data from these encounters are fed back into the system to refine risk algorithms and ensure responsive, personalized financial support. | AI-driven decision support tools that personalize lifestyle interventions—such as nutrition, physical activity, and psychosocial support—based on patient characteristics, treatment history, and readiness for change. The system delivers tailored recommendations and initiates referrals or resource connections automatically, while preserving patient autonomy through opt-in options and non-intrusive communication. Engagement and outcomes are continuously monitored to refine intervention strategies over time. | Machine learning algorithms analyze clinical, demographic, and behavioral data to identify patients at risk for emerging social challenges—such as housing insecurity, food insecurity, or job loss—before these issues affect health outcomes. The system provides timely, personalized alerts and support options, enabling care teams and patients to intervene early. Proactive outreach is designed to inform and empower patients without creating alarm, and feedback loops help refine risk prediction over time." | Predictive models analyze patient-reported symptoms, biometric data, and sensor inputs to anticipate emerging symptom burden and initiate earlier interventions. These models guide timely specialty referrals and adaptive symptom management strategies, supported by real-time care team response. Continuous feedback from wearables and patient communications informs personalized care plans and drives iterative refinement of symptom management protocols. |
| Level 4 Reportable  Definition:  Care processes are fully standardized, implemented across all relevant settings, and systematically documented in ways that support institutional reporting, quality monitoring, and accountability. Leadership regularly reviews data to guide training, refine workflows, identify disparities, and inform resource allocation. | Institutional dashboards track adherence to survivorship care planning, highlighting care gaps and variation in documentation and follow-up. Leadership uses this data to monitor compliance, guide training efforts, and assess implementation across departments. | Routine institutional reports track adherence to cancer-specific follow-up screening guidelines and identify patients overdue for surveillance. Dashboards support monitoring of no-shows, cancellations, and reminder effectiveness. Leadership uses these data to guide outreach, assess disparities, and refine care coordination strategies across clinics | Dashboards monitor referral completion rates, provider response times, and timeliness of specialty care delivery. Institutional goals for referral performance are defined, tracked, and used to guide quality improvement efforts. Leadership reviews these data to ensure high-priority patients receive coordinated and timely access to specialty services. | Financial hardship trends are monitored at the institutional level, enabling leadership to identify disparities, refine financial assistance strategies, and allocate resources effectively. | Reports and dashboards track patient engagement with lifestyle interventions—such as nutrition counseling, physical activity programs, and behavioral health services—enabling the institution to monitor referral consistency and identify gaps in service utilization. These data are used to implement targeted improvements, and direct-to-patient screening tools further support identification of needs. | Leadership reviews institutional data on social and practical challenges—such as food insecurity, transportation, and housing instability—to identify high-risk populations. This data informs resource allocation and guides improvements in access to supportive services across the system. | Data dashboards monitor symptom burden trends across the patient population, drawing from sources such as patient-reported outcomes and message analytics. Symptoms are categorized by type and clinical specialty, allowing for targeted improvements in specialty care access, triage workflows, and symptom management protocols. Leadership uses this data to guide resource allocation and identify unmet needs. |
| Level 3 Unified  Definition:  Workflows and tools are standardized and consistently implemented across clinics or providers. Processes are embedded in the EHR, and team roles are clearly defined. Care delivery is routine and proactive, supported by established communication pathways between disciplines. While practices are consistent, monitoring and feedback mechanisms remain limited. | Survivorship care plans are systematically recorded in the EHR using structured templates that include treatment summaries, follow-up schedules, and preventive care (e.g., vaccine referrals). Plans are accessible to the full care team, including primary care providers, and supported by communication channels between oncology and primary care. Roles are clearly assigned for survivorship planning, and dedicated personnel—such as medical assistants or patient navigators—support documentation and implementation | Screening for recurrence and new primary cancers is integrated into the EHR, with most patients automatically scheduled for routine follow-ups based on cancer-specific guidelines. Communication between oncology and primary care supports coordination of orders and referrals. Where provider-entered orders are required, streamlined workflows support timely scheduling. | Clear referral procedures are understood by all stakeholders.  EHR-integrated workflows use structured templates and clinical triggers to prompt timely referrals, such as lymphedema or psychosocial distress. Care team roles in initiating and managing referrals are clearly defined and implemented. Referrals are proactive and patient-centered, with assigned teams (e.g., psychology, social work) responsible for follow-up. Communication between oncology and primary care supports coordination, and referral processes follow clearly established institutional protocols. | Financial toxicity screening is embedded in the EHR and administered consistently across clinical settings. Structured referral pathways connect eligible patients to financial navigators or assistance programs. Roles and responsibilities for screening, referral, and follow-up are clearly defined among the care team, ensuring that financial concerns are identified and addressed in a timely, standardized manner | Referrals for lifestyle-related services—such as nutrition counseling, behavioral health, and exercise programs—are documented in the EHR and accessible to patients. Providers use structured tools to assess lifestyle behaviors and guide referrals. Patient navigators and Cancer Resource Coordinators support education and follow-up, with clearly defined roles across the care team for screening, documentation, and coordination. | Social determinants of health (SDOH) screening is embedded in patient assessments and used to identify needs such as food insecurity, transportation challenges, and housing instability. Structured referral pathways connect patients to case managers or community resource coordinators, with some referrals initiated automatically through the EHR. Roles for screening, referral, and follow-up are clearly defined across the care team. Patients have access to supportive resources, including transportation assistance (e.g., metro cards, gas cards, rideshare services) and temporary housing options. | EHR-integrated tools support standardized assessment and tracking of physical symptoms such as pain, fatigue, and neuropathy. Clinician-facing decision support guides documentation and referrals to specialty care. Patient-reported symptoms are triaged to the appropriate team member for timely follow-up. Dedicated survivorship clinics support long-term symptom management and continuity of care. |
| Level 2 Planned  Definition:  Basic workflows, SOPs, or screening tools have been introduced but are not yet consistently applied. Care delivery is guided by foundational policies or tools, but implementation varies, and roles are not clearly defined. These efforts rely heavily on individual clinician initiatives and are not yet embedded in routine practice. | Standard operating procedures for survivorship care are in place, supported by EHR prompts and dot phrases for selecting malignancies aligned with clinical guidelines. These tools encourage care planning discussions and documentation, but adherence remains variable. Basic educational materials are available to patients. | Basic workflows for follow-up screenings are established, including EHR-based scheduling processes and overdue screening notifications. Cancer-specific guidelines inform practices, but implementation depends on clinicians reviewing results, ordering appropriate tests, and coordinating with patients or clinics. | Referral checklists and workflows are available to support specialty care referrals, and some care team members receive training in survivorship-specific referral processes. Screening tools may inform these referrals, but usage and scope are inconsistent. There is no centralized system for tracking referral completion or coordination across teams. | Standardized screening for financial hardship has been introduced, and educational materials on financial assistance are available. Social workers may provide support when involved, but referrals depend on clinician initiative. Financial counselor availability varies, and there is no integrated process for systematically connecting patients to financial resources. | EHR prompts and standard questionnaires are used to encourage discussions about lifestyle behaviors such as diet, physical activity, and smoking cessation. Educational materials and pathways to supportive health services (e.g., case management) are available, but use of these supports is inconsistent across clinicians. | Basic screening for practical and social concerns—such as transportation, food insecurity, and housing—is in place. Referral pathways to case management or community resource coordinators exist, but follow-up is inconsistent and care team roles in initiating and managing referrals remain unclear. | Standardized symptom checklists for physical effects—such as pain, fatigue, and neuropathy—have been introduced, and some symptoms are tracked between visits. While clinicians may initiate referrals to specialty care based on reported symptoms, processes are not automated and vary across settings. Patient-reported outcomes, if collected, are not yet fully integrated into routine workflows, limiting their use in clinical decision-making. |
| Level 1 Ad Hoc  Definition:  Care processes are inconsistent, unstructured, and highly variable, typically dependent on individual clinician preferences or local team norms. There are no standardized tools, workflows, or documentation practices in place. Patient access to services is uneven and often reliant on personal advocacy or provider initiative. | Survivorship care planning is inconsistent, often driven by individual clinician preferences. While some patients receive care plans, documentation and follow-up are not standardized | Follow-up screenings for recurrence and new cancers are inconsistent, often depending on patient initiative and treating oncologist recommendations, which can lead to gaps in care. | Referrals are informal and highly clinician-dependent, with no standardized tracking system. Access to specialty care (e.g., genetic testing), often hinges on individual provider knowledge and professional networks, resulting in inconsistent referral practices  Referrals are informal and highly clinician-dependent, with no standardized tracking system. Access to specialty care, including genetic testing, often hinges on personal networks and individual provider knowledge, resulting in inconsistent referral practices | Although educational materials on financial assistance are available, there is no formal screening or referral process for financial hardship. Patients must initiate the conversation, after which the responsibility falls to the physician to connect them to appropriate resources the physician may not be fully aware of. This results in inconsistent identification and support for financial concerns. | Discussions about lifestyle behaviors—such as diet, physical activity, and smoking cessation—are sporadic and typically occur during clinical encounters without consistent documentation or follow-up. While some providers refer patients to services like nutrition counseling or psychosocial support, there is no standardized process for connecting patients to supportive health services such as case management or resource navigation. Utilization depends heavily on individual clinician knowledge and initiative. | Practical and social concerns—such as transportation, employment, and social isolation—are not consistently identified or addressed. Referrals to social work are uncommon, in part because a dedicated social worker is not consistently available. | Clinicians assess physical side effects—such as pain, fatigue, or neuropathy—primarily during routine clinic visits, without a standardized protocol or systematic use of patient-reported outcomes (PROs). As a result, symptom monitoring is variable, and less common or sensitive issues (e.g., sexual dysfunction) may be overlooked. |

Supplemental Table 2. Focus-Group Incremental Modifications:

Red: Focus Group 1, Purple: Focus Group 2, Green: Focus Group 3, Light Blue: Focus Group 4, Orange: Focus Group 5

|  | Survivorship Care Planning | Risk of Recurrence or New Cancers | Care Referrals | Financial Hardship/Toxicity | Lifestyle Behaviors & Supportive Health Services | Practical and Social Effects | Physical Effects |
| --- | --- | --- | --- | --- | --- | --- | --- |
| Level 5 Optimized | Advanced analytics provide real-time monitoring of survivorship care plan completion. AI-driven alerts ensure personalized, proactive care planning. Leadership continuously refines workflows based on patient outcomes and emerging best practices. | AI-driven analytics identify high-risk patients for recurrence, enabling personalized surveillance. Screening intervals and outreach strategies are dynamically adjusted based on real-time data.  -HIE pushes outside system procedures  Doesn’t exist in practice yet | Predictive analytics anticipate referral needs based on patient profiles. ~~The system proactively generates referrals before symptoms escalate, optimizing resource allocation.~~ | Real-time dashboards monitor financial distress in cancer patients at routine clinic visits, triggering proactive financial counseling interventions before economic hardship affects treatment adherence. | AI-driven decision support tools personalize lifestyle interventions based on patient characteristics, ensuring tailored recommendations for nutrition, exercise, and psychosocial support.; Creating interventions; automated process of implementing referrals and resources to appropriate patients Without being intrusive | Machine learning algorithms identify patients at risk for social challenges (e.g., housing insecurity, job loss), allowing for proactive intervention before issues impact health outcomes.; Giving patients a “heads up” regarding what could happen | Predictive models analyze symptom data to anticipate patient needs, allowing for earlier interventions, optimized specialty referrals, and proactive symptom management strategies. Feedback: -wearables, sensors -response of care team |
| Level 4 Reportable | Institutional reports track adherence to survivorship care planning, highlighting ~~care gaps~~ and variations in follow-up. Dashboards allow leadership to analyze compliance and guide training efforts.  -cancer survivorship clinic  -this exists but due to the “extra work” and no funds, many places abandoned trying to accomplish it | Regular reports track adherence to follow-up screening guidelines and identify patients overdue for screenings. Leadership uses this data to target outreach.; -no show/cancellation follow-up within the institution -dashboards: accurately reporting -exists but not always accurate. Patients do get “reminders” if overdue for screening but if they had mastectomy, imaging outside or metastatic its accurate | Dashboards track referral completion rates ~~and provider response times, ensuring that high-priority patients receive specialty care in a timely manner.~~; -Specify goals & track them | Financial hardship trends are monitored at the institutional level, allowing leadership to refine financial assistance strategies and allocate resources effectively.; -this is not done, but it would be nice | Reports analyze engagement with lifestyle interventions, ensuring consistent referrals and identifying gaps in utilization of supportive services. -ongoing metrics & analysis of use of referrals & resources, and implementation of changes to improve -Direct to patient screening | Leadership reviews data on social and practical challenges, identifying high-risk populations and adjusting resource allocation to improve access to services. | Data dashboards monitor symptom burden trends across the patient population, allowing for targeted improvements (and resource allocation) in specialty care access and symptom management protocols. -analytics on messages -categorizing of messages -individualized to specialty |
| Level 3 Unified | Survivorship care plans are systematically recorded in the EHR and accessible to the entire care team. Structured templates ensure inclusion of key elements, such as treatment summaries and follow-up schedules.; -including specific note or tab -PCP & oncologist communication channel -extra time allowed for survivorship discussion -communication between teams -team assigned roles for survivorship cases in PCP -vaccine referrals -check list for specific points -exists but takes manpower/time to create this & we need resources to do the “work” or have it auto-generated. It’s essentially an unfunded recourse. We have places in EHR/templates already -resource/time provided for care can be MA, patient navigator, etc. | Screening for recurrence and new cancers is fully integrated into the EHR. Most patients are (~~automatically~~) scheduled for routine follow-ups. Coordination (communication) between oncology (and primary care improves.); Input from primary care orders/referrals -Prioritized orders (imaging); no shows/cancellation fall off list -This would be amazing, but there is a barrier -barrier = requires in state of TX “orders,” which must be entered by providers -solution: @ time of mammogram result, “verbal order” by provider & scheduled @ that time | ~~EHR-integrated referral processes ensure automatic documentation of specialty referrals.~~ Triggers in the system prompt referrals based on patient needs (e.g., lymphedema, psychosocial distress).; Template -Role is clear and implemented -referrals proactive to patient -teams assigned on specific referrals like psychology, social work, etc. -communication between PCP & oncologist -clear guidelines of processes | Financial toxicity screening is embedded in the EHR, with structured referrals to financial navigators and assistance programs for eligible patients.; -and role clarity | Lifestyle and supportive service referrals are now documented in the EHR (MyChart), with structured pathways for patients to access dieticians, behavioral health specialists, and exercise programs. -in training, use of EPIC SDOH Lifestyles/Behaviors: tool by providers to then decide on needed referrals and resources -Patient Navigators -Screening for food insecurity: through QR codes in patient rooms? -tool for screening for risks -nutrition tools available -Cancer Resource Coordinator distribution available; (might need to be further refined and/or include other services) | Social determinants of health (SDOH) screening is embedded in patient assessments (who does this? AI??), with automated referral pathways to case managers and community resources.; -Role Clarity -Alternative transport methods: metro card, gas cards, uber account. -Referrals: auto method -some of this already exists at Harris Health -temporary housing options available | EHR-integrated (this can be smarter with trending etc.) clinician support tools guide standardized assessment of physical effects, ensuring routine symptom tracking and specialty referrals.; -difficult to do –integrated w/ EHR -messages triaged to correct person and timely answered -establishment of a long-term survivor clinic |
| Level 2 Planned | Standard operating procedures (SOPs) exist for survivorship care, ensuring some consistency. ~~EHR prompts~~ SOPs encourage clinicians to discuss care plans, but adherence varies. Basic educational materials are available.; -Dot phrases in EPIC: available for specific malignancies according to guidelines; check list for care team | The institution establishes basic workflows for follow-up screenings, including scheduling processes and overdue screening notifications in the EHR (and Mychart?). However, monitoring is still clinician-dependent.; -Depends on what screening; this is cancer specific -we have set guidelines/ways to do screening, but it relies on providers reading reports, ordering tests, & tests being scheduled by patients/clinics | Referral checklists exist for specialty care, but completion depends on clinician adherence. There is no centralized tracking or coordination across teams.; -training of care team to implement on survivorship -process for referring -screening tool for referrals, beyond Edmonton for other referrals | Standardized financial toxicity screening is introduced, but it relies on clinician initiative. Educational materials on financial assistance are available.; Financial counselor availability -we need a tool like this and automatic referrals -social worker is available w/ resources | EHR prompts encourage discussions on lifestyle behaviors, and general educational materials are provided to patients, though implementation remains inconsistent.; -standard questionnaires given at each clinic visit -case manager available -resource pathway exists -clear pathways could help all clinicians implement/utilize the available resource at Harris Health | Basic screening for social and practical challenges (e.g., transportation, work) is introduced, but follow-up is inconsistent.; -Role is unclear (who is doing this: MD, RN, SW, MA?) -Clear pathways with facilitators. Referrals to CM & CRC driven by provider | Standardized symptom checklists for physical effects (e.g., pain, fatigue) are introduced, but referrals to specialty care are not automated.; -Recorded & tracked communication between visits -Symptoms are managed & addressed on an individual basis (do we want them automated?) (if PROs are collected, they may not be in a place where clinicians can use them) |
| Level 1 Ad Hoc | Survivorship care planning is inconsistent, relying on individual clinician preferences. Some patients receive care plans, but there is no standardized documentation or follow-up. | Follow-up screenings for recurrence and new cancers occur inconsistently. Patients often rely on their own initiative to schedule follow-ups, leading to gaps in care.; Plus, recommendations of treating oncologists | (Referrals are informal and clinician-dependent, with no standardized tracking. Some patients receive specialty referrals, while others do not.; -knowing who to see “know people” -referral for genetic testing) | Financial concerns are only addressed if a patient brings them up. There is no formal assessment or referral process for financial hardship and; no financial counselor on site ” Who to go to?” -this is how it is and we engage social worker/financial advisor -patient initiated conversation | Discussions on lifestyle modifications (e.g., nutrition, physical activity, mental health) are sporadic, with no systematic documentation or referrals.; -discussion during clinical encounter with possible documentation or orders places -options to refer -document discussions -options to refer to nutritionist -patient portal | Practical and social concerns (e.g., transportation, employment, social isolation) are not consistently addressed, and referrals to social work are uncommon.; -no consistent social worker on call | Clinicians assess physical side effects such as pain, fatigue, or neuropathy on an ad hoc basis. No formal screening protocols exist.  -wait until next clinic visit  (inconsistent in absence of routine USC of PRO’s)  (less common symptoms or scarier symptoms (sexual dysfunction) if addressed at all) |

Supplemental Table 3. Post-Focus Group Model:

After receiving feedback from the iterative focus groups, the Cancer Survivorship Maturity Model (CSMM) was modified by the authors to reflect specific changes made by the participants or general themes repeated by several participants. Terminology was modified to match the clinicians’ understanding and the model was overall simplified by removing statements which focus group members felt were redundant or would not apply in a real-world context. Several statements were moved from one level to another to match the clinical workflows. This model was built in a redcap survey to enable model evaluation within the NCISS Awardee community.

|  | **Survivorship Care Planning** | **Risk of Recurrence or New Cancers** | **Care Referrals** | **Financial Hardship/Toxicity** | **Lifestyle Behaviors**  **& Supportive Health**  **Services** | **Practical and Social Effects** | **Physical Effects** |
| --- | --- | --- | --- | --- | --- | --- | --- |
| **Level 5 Optimized** | Personalized, proactive care planning performed.  Closed loop data monitoring of survivorship care plan completion enables leadership to continuously refine workflows based on patient outcomes and emerging best practices. | Anticipatory identification of high-risk patients for recurrence, enabling personalized surveillance. Screening intervals and outreach strategies are dynamically adjusted based on real-time data feedback. | Anticipatory needs-based referrals personalized to individual risk, preferences and patient context. The system proactively generates referrals before symptoms escalate, optimizing resource allocation. | Financial distress monitoring is integrated into clinical workflows, triggering proactive financial counseling interventions before economic hardship affects treatment adherence. | Patient data and preferences feed back into the system offering personalized lifestyle interventions based on patient characteristics, ensuring tailored recommendations for nutrition, exercise, and psychosocial support. | Closed loop data feedback within system identifies patients at risk for social challenges (e.g., housing insecurity, job loss), allowing for proactive intervention before issues impact health outcomes. | Personalized analysis of symptom data anticipates patient needs, allowing for earlier interventions, optimized specialty referrals, and proactive symptom management strategies. |
| **Level 4**  **Reportable** | Institutional reports track adherence to survivorship care planning, highlighting care gaps and variations in follow-up. Dashboards allow leadership to analyze compliance and guide training efforts. | Data dashboards monitor symptom burden trends across the patient population, allowing for targeted improvements in specialty care access and symptom management protocols. | Dashboards track referral completion rates and provider response times, ensuring that high-priority patients receive specialty care in a timely manner. | Financial hardship trends are monitored at the institutional level, allowing leadership to refine financial assistance strategies and allocate resources effectively. | Reports analyze engagement with lifestyle interventions, ensuring consistent referrals and identifying gaps in utilization of supportive services. | Leadership reviews data on social and practical challenges, identifying high-risk populations and adjusting resource allocation to improve access to services. | Data dashboards monitor symptom burden trends across the patient population, allowing for targeted improvements in specialty care access and symptom management protocols. |
| **Level 3**  **Unified** | Survivorship care plans are systematically recorded in the EHR and accessible to the entire care team. Structured templates ensure inclusion of key elements, such as treatment summaries and follow-up schedules. | Screening for recurrence and new cancers is fully integrated into the EHR. Most patients are automatically scheduled for routine follow-ups. Coordination between oncology and primary care improves. | EHR-integrated referral processes ensure automatic documentation of specialty referrals. Triggers in the system prompt referrals based on patient needs (e.g., lymphedema, psychosocial distress). | Financial toxicity screening is embedded in the EHR, with structured referrals to financial navigators and assistance programs for eligible patients. | Lifestyle and supportive service referrals are now documented in the EHR, with structured pathways for patients to access dieticians, behavioral health specialists, and exercise programs. | Social determinants of health (SDOH) screening is embedded in patient assessments, with automated referral pathways to case managers and community resources. | EHR-integrated clinician support tools guide standardized assessment of physical effects, ensuring routine symptom tracking and specialty referrals. |
| **Level 2**  **Planned** | Standard operating procedures (SOPs) exist for survivorship care, ensuring some consistency. EHR prompts encourage clinicians to discuss care plans, but adherence varies. Basic educational materials are available. | The institution establishes basic workflows for follow-up screenings, including scheduling processes and overdue screening notifications in the EHR. However, monitoring is still clinician dependent. | Referral checklists exist for specialty care, but completion depends on clinician adherence. There is no centralized tracking or coordination across teams. | Standardized financial toxicity screening is introduced, but it relies on clinician initiative. Educational materials on financial assistance are available. | EHR prompts encourage discussions on lifestyle behaviors, and general educational materials are provided to patients, though implementation remains inconsistent. | Basic screening for social and practical challenges (e.g., transportation, work) is introduced, but follow-up is inconsistent. | Standardized symptom checklists for physical effects (e.g., pain, fatigue) are introduced, but referrals to specialty care are not automated. |
| **Level 1**  **Ad Hoc** | Survivorship care planning is inconsistent, relying on individual clinician preferences. Some patients receive care plans, but there is no standardized documentation or follow-up. | Follow-up screenings for recurrence and new cancers occur inconsistently. Patients often rely on their own initiative to schedule follow-ups, leading to gaps in care. | Referrals are informal and clinician-dependent, with no standardized tracking. Some patients receive specialty referrals, while others do not. | Financial concerns are only addressed if a patient brings them up. There is no formal assessment or referral process for financial hardship. | Discussions on lifestyle modifications (e.g., nutrition, physical activity, mental health) are sporadic, with no systematic documentation or referrals. | Practical and social concerns (e.g., transportation, employment, social isolation) are not consistently addressed, and referrals to social work are uncommon. | Clinicians assess physical side effects such as pain, fatigue, or neuropathy on an ad hoc basis. No formal screening protocols exist. |
